# The obtaining of high-density specimens and analysis of mechanical strength characteristics of a composite based on ZrO_2_-WC nanopowders

**DOI:** 10.1186/1556-276X-9-355

**Published:** 2014-07-15

**Authors:** Edwin Gevorkyan, Olga Melnik, Vladimir Chishkala

**Affiliations:** 1 Mechanical Department, Ukrainian State Academy of Railway Transport (UkrSART), Feyerbakh square 7, Kharkov 61001, Ukraine; 2 Department of Physics and Technology, V.N. Karazin Kharkiv National University, Kurchatov Av. 31, Kharkov 61108, Ukraine

**Keywords:** Electroconsolidation, ZrO_2_-WC, Hardness, Fracture toughness

## Abstract

The structures, processes of shrinkage, and phase composition of the compact system ZrO_2_-WC, obtained by hot pressing with the transmission of high current, are considered in the article. We found that as a result of compaction, the ZrO_2_-WC-ceramics have uniform density distribution, with the following optimal mode consolidation values *T* = 1,350°C, *P* = 30 MPa and *t* = 2 min. These conditions allow us to achieve the best combination of ceramic properties by criteria density and strength.

## Background

Recently, most binary systems were made based on ZrO_2_ such as ZrO_2_-TiB_2_, ZrO_2_-TiCN, ZrO_2_-SiC, ZrO_2_-TiN, and ZrO_2_-TiC. Consequently, high mechanical properties of the material can be expected when ZrO_2_ is hardened by nanoparticles of the second phase (tungsten carbide). It will allow extensive use of obtained ceramics.

It is known that tungsten carbide is widely used in the manufacture of hard alloys based on WC-Co due to its high resistance to wear and low temperatures during use. However, the thermal stability of the cobalt binder greatly limits its use as a structural component, where high heat resistance, resistance to oxidation, and corrosion are very important. Previously, attention was paid to determine the optimum ZrO_2_ in the composite materials based on WC made by high-energy FAST methods [1,2]. Also, the authors in [3] reported that the addition of 30% micron-sized WC to ZrO_2_-matrix significantly increases the hardness and fracture toughness, but their values were low.

Research on the possibility of compacting ZrO_2_-WC composites via hot pressing with electric current (electroconsolidation) is the purpose of this work. It is also important to identify optimal regimes to obtain high-density samples having homogeneous microstructure with high mechanical characteristics.

## Methods

The nanopowders were mixed using a planetary milling plant ‘Pulverisette 6’(Fritsch GmbH, Idar-Oberstein, Germany with isopropyl alcohol for 2 h for a uniform distribution of particles in the sample. The rotation speed of planetary disk is 160 rpm. To break the agglomerates, alumina milling balls were added to the container.

Installation for hot vacuum pressing, designed and patented by the authors, was done to consolidate the powders. This installation, in comparison with the well-known FAST method in Europe, differs mainly because of the possibility that it uses a conventional AC power frequency without special optional equipment pulse generators. This method later in this article will be referred to as electroconsolidation.

The nanopowders were sintered using a hot pressing facility with a direct current under a pressure of 30 MPa and held for 2 min at various temperatures. Further studies were done on molded samples such as tablets of 20 mm in diameter. Sintering curve looks like this: at a pressure of 10 MPa, the temperature was raised at 150°C/min up to a temperature of 600°C; then, at the same pressure, the temperature was adjusted to a holding temperature (1,200°C to 1,400°C). This temperature was held for 2 min. At the same time, the pressure was raised to 30 MPa. After the rise of the holding temperature stopped, the sample cooled and formed. Pressure is removed after the final cooling. Full-time consolidation was 15 min.

The microstructure of the nanoceramic compositions, obtained by electroconsolidation, was examined by scanning electron microscopy; by the same method, the grain sizes of the obtained samples were evaluated. The samples for electron microscopic studies were prepared as shear of sintered tablets.

Using a universal hardness tester, the Vickers hardness (HV_10_) of the composite is evaluated with a load of 10 kg. The fracture toughness (*K*_IC_) calculations were made based on the measurements of the radial crack length produced by Vickers (HV_10_) indentations, according to Anstis formula [4]. The reported values are the averages of the data obtained from five indentation tests.

Detailed microstructural characterization and phase identification were carried out using a Quanta 200 3D (FEI Co., Hillsboro, OR, USA) scanning electron microscope (SEM) and a Rigaku Ultima IV X-ray diffractometer (Rigaku Europe SE, Ettlingen, Germany) (CuKα radiation, Ni filter).

## Results and discussion

The commercially available high-purity WC (primary crystallite size 30 nm, Wolfram, Salzburg, Austria) and ZrO_2_ (3 mol% Y_2_O_3_) powders (primary crystallite size 20 nm, The Research Centre of Constructional Ceramics and The Engineering Prototyping, Russia) were used as starting powders. The sintering parameters and relative density of the obtained ZrO_2_-WC composites are presented in Table 1.

**Table 1:** **The sintering parameters and relative density of the obtained ZrO**_**2**_**-WC composites**

**Material composition**	**Sintering temperature (°C)**	**Holding time (min)**	**wt.% WC**	**Relative density (%)**
Z10WC	1,250	2	10	96.7
	1,250	4		96.8
	1,300	2		97.3
	1,350	2		98.5
Z20WC	1250	2	20	98.3
	1,250	4		98.5
	1,300	2		99.3
	1,350	2		99.5
Z30WC	1,250	2	30	96.5
	1,250	4		96.9
	1,300	2		95.0
	1,350	2		97.3

Table 1 shows that the holding time is a temperature-independent parameter and slightly influences the densification. The density data reveal that the maximum density of approximately 99.5% *ρ*_th_ can be achieved in composite sintered at 1,350°C and holding time of 2 min with 20 wt.% WC additive.

**Figure 1:**
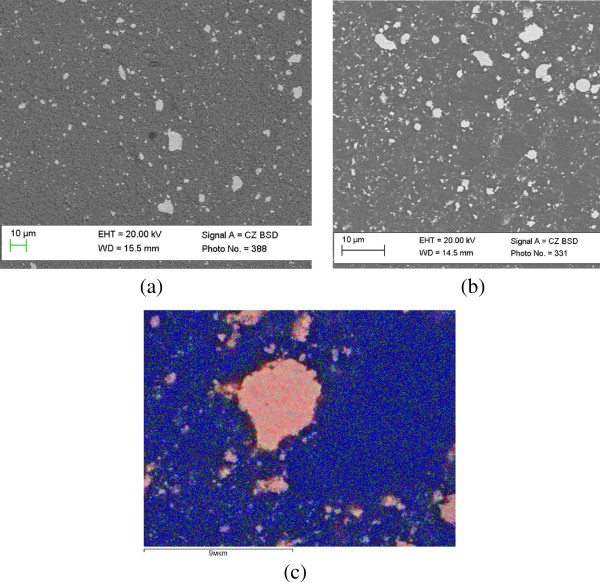
**The ZrO**_**2**_**-WC composite microstructure in the different regimes.** SEM-SE image of the composite microstructure based on ZrO_2_ with 10 wt.% **(a)** and 20 wt.% **(b)** WC and SEM images ZrO_2_-WC ceramics in regime CCL **(c)**.

**Figure 2:**
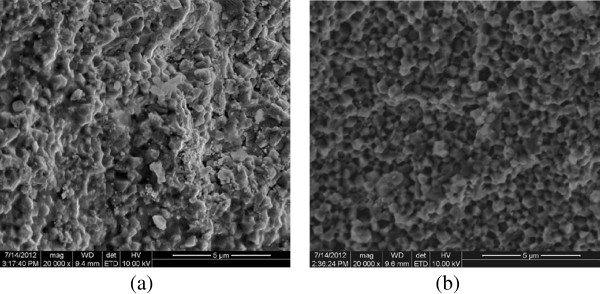
**SEM-SE image of the microstructures of ZrO**_**2**_**-20 wt.% WC.** WC was sintered at *T* = 1,350°C and *P* = 30 MPa during the holding time **(a)** and *T* = 1,350°C and *P* = 30 MPa applied in the beginning of the sintering cycle **(b)**.

Microstructure of ZrO_2_-WC composites with 10% and 20% WC is shown in Figure 1. The WC phase (bright) was uniformly dispersed in the ZrO_2_-matrix (dark) except for a number of agglomerated particles. However, a careful study using computerized color cathodoluminescence (CCL) attached to the SEM allowed for the determination of a significant amount of zirconia particles in the light phase (Figure 1a). This fact indicates a rather homogeneously mixed ZrO_2_-WC composition.It was found that the maximum pressure is only applied when the compact system reach its maximum temperature (for complete degassing of adsorbed gases). This mode results in the formation of finer structure of material (Figure 2a), in which the pressure was applied at the beginning of the sintering cycle and was remained constant (Figure 2b). The application of the maximum pressure at lower temperatures results in an increased porosity due to the presence of adsorbed gases. Shrinkage due to the evaporation of absorbed moisture and burnt impurities competes with the process of thermal expansion in the first stage of the sintering process.

**Figure 3:**
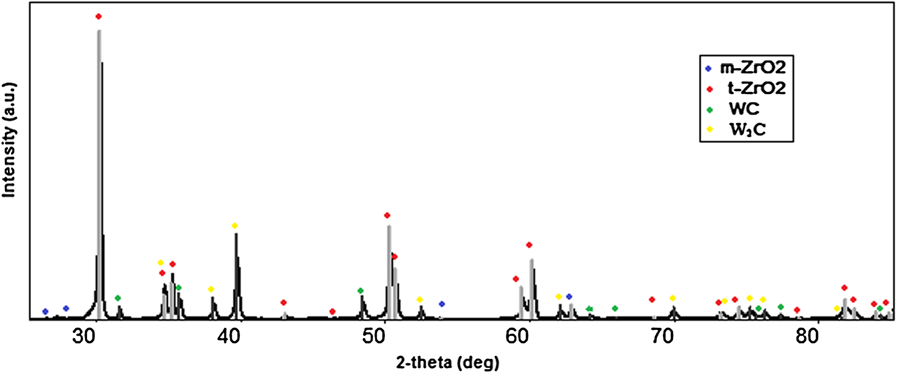
**XRD patterns of polished cross-sections of the ZrO**_**2**_**-20 wt.% WC composites.***T* = 1,350°C, *P* = 30 MPa, and holding time = 2 min.

**Figure 4:**
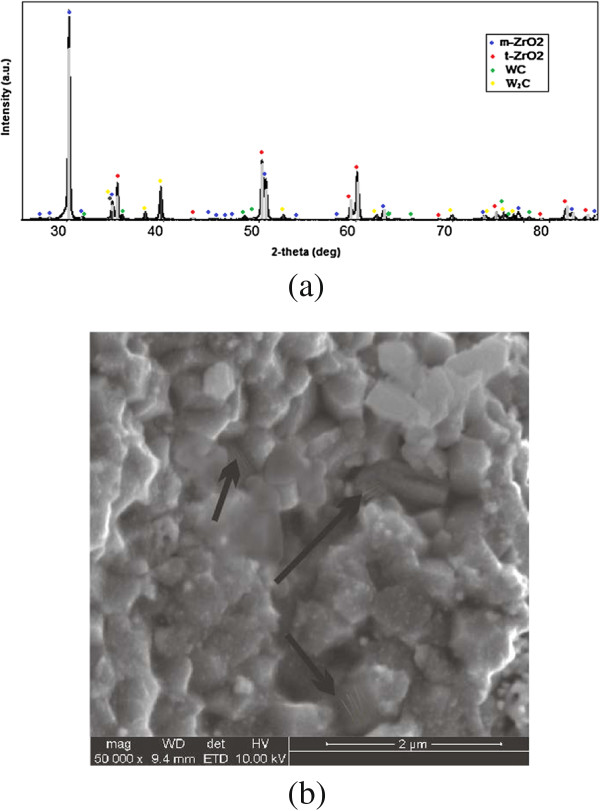
**XRD patterns (a) and SEM-SE image of microstructure (b) of fractured surfaces of the ZrO**_**2**_**-20 wt.% WC composites.***T* = 1,350°C, *P* = 30 MPa, and holding time = 2 min.

Moreover, the high purity of the starting powder and narrow particle size distribution were the cause of avoidance of abnormal growth (exceeding some medium-sized grains) and the homogeneity of the material microstructure. The latter circumstance is also characterized by a uniform distribution of density and, accordingly, the diameter of the microhardness indentation of the sample that allows to obtain materials with high mechanical properties and longer service life extension of ceramic products. The most uniform hardness distribution on the diameter of the sample was indicated in ZrO_2_-20 wt.% WC that was sintered at 1,300°C and with a pressure of 30 MPa with a holding time of 2 min.Figure 3 shows the X-ray of the polished surface, and Figure 4a shows the X-ray of the fracture pattern and of the samples. The increasing number of monoclinic zirconium oxide peaks indicates that there is a tetragonal-monoclinic transformation during loading. The average grain size of the sample is 350 nm. The structure is homogeneous and contains no grains with sizes that differ greatly from the others. That is, the addition of 20 wt.% tungsten carbide further hardened the material based on zirconium oxide, while it demonstrated the abnormal grain growth and formation of a fine structure with a high content of tetragonal phase which is able to transform into the monoclinic phase (under the influence of stress) in the vicinity of the crack tip.

The microstructure of fracture surfaces of ceramics obtained at 1,350°C. One can distinguish two types of areas: areas of intergranular fracture and the so-called twinning topography (Figure 4b indicated by arrow). The paper [5] stated that the presence of fracture surface areas with relief twinning can indicated that the structure undergoes a stress-induced martensitic (tetragonal-monoclinic) transformation during fracture. We assume that some of the grains with twin structure are zirconia grains. However, to confirm this hypothesis, the chemical analysis of the samples should be carried out.

The formation of W_2_C assumed to be a reaction between ZrO_2_ and WC [6]:

(1) $$\begin{array}{c} {\mathrm{ZrO}}_{2}-x+\;2y\mathrm{WC}\;=yW_{2}C\;+\;{\mathrm{ZrO}}_{2}-x-y\\ \;+y\mathrm{CO}, \end{array}$$

where *x* is the oxygen vacancy concentration in the ZrO_2_ as a result of the dopant concentration, and *y* is the additional vacancy concentration created in the ZrO_2_ due to the reaction with WC.

This reaction contributes to the formation of additional oxygen vacancies and W_2_C. The occurrence of additional oxygen vacancies leads to an increase of non-stoichiometry ZrO_2_ phase. This can improve the diffusion coefficient in a certain degree, whereby the mass transfer occurs quickly and, therefore, increases the rate of sintering.

**Figure 5:**
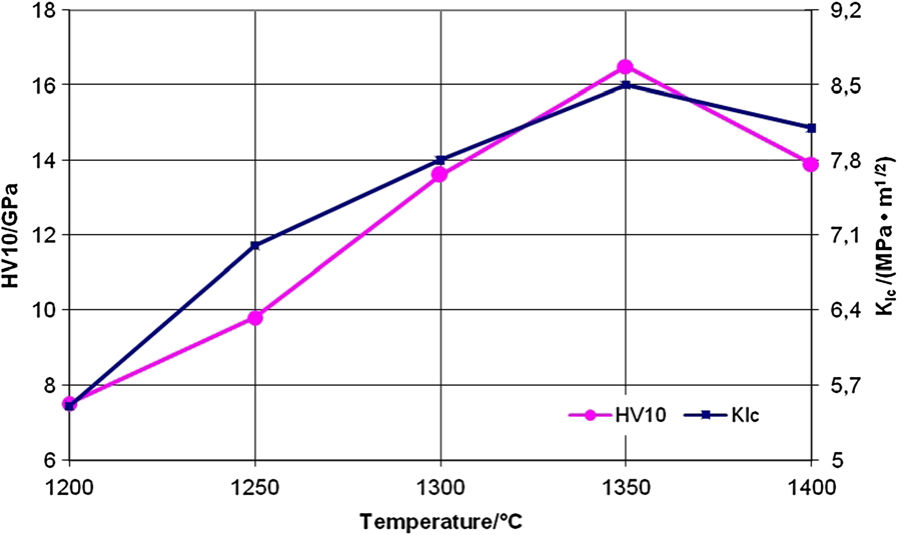
**Vickers hardness and fracture toughness of the ZrO**_**2**_**-20 wt.% WC composites.** Vickers hardness and fracture toughness as functions of the sintering temperature.

The Vickers hardness (HV_10_) and indentation fracture toughness (*K*_IC_) of the ZrO_2_-20 wt.% WC composites are graphically presented as a function of the sintering temperature in Figure 5.

The hardness variation with sintering temperature is closely related to the bulk density and microstructural features. The hardness increased continuously with increasing temperature from 1,200°C to 1,350°C (Figure 5), due to an increased densification, reaching a maximum hardness at full densification when temperature was at 1,350°C. At higher sintering temperatures, the hardness slightly decreased due to the increased WC and ZrO_2_ grain size, as well as the partial spontaneous transformation of the ZrO_2_ phase.

The fracture toughness increased rapidly from 5.5 to 8.5 MPa m^1/2^ with increasing temperature from 1,200°C to 1,350°C (Figure 5), followed by a decreasing trend to 8.1 MPa m^1/2^ at 1,400°C. The high value of fracture toughness may be due to the fact that a part of the tetragonal phase of ZrO_2_ transforms to the monoclinic ZrO_2_ (Figure 4) during electroconsolidation at a temperature of 1,350°C.

**Figure 6:**
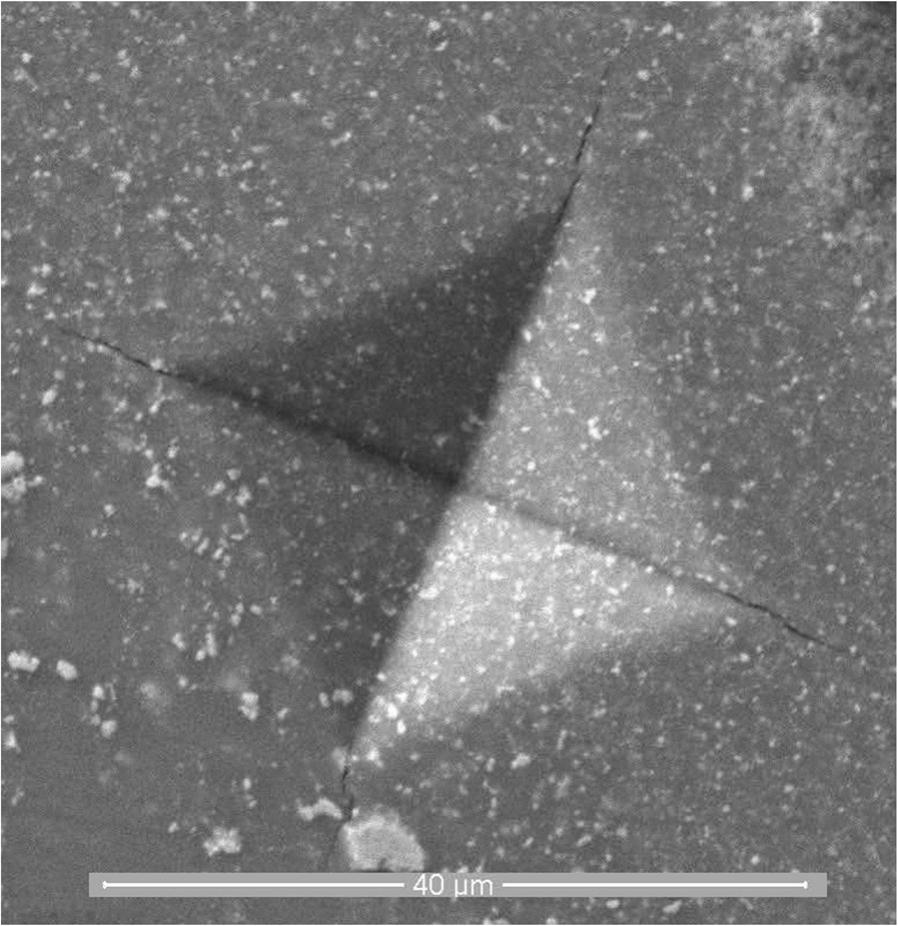
**SEM-SE microstructure of fracture surface of WC-ZrO**_**2 **_**composite.***T* = 1,350°C, *P* = 30 MPa, and holding time = 2 min.

Moreover, in the ZrO_2_-WC composites, crack deflection is an effective toughening mechanism besides the ZrO_2_ phase transformation toughening. The radial crack pattern originating in the corners of the Vickers indentations revealed that the propagating cracks were deflected by the WC grains (Figure 6), which was also observed in hot pressed ZrO_2_-WC composites [5].

## Conclusions

Electroconsolidation provides a uniform density distribution, without any plasticizers that are potential sources of impurities and additional porosity in the sintered product. The maximum density of the ZrO_2_-20 wt.% WC composite was obtained at 1,350°C for 2 min at 30 MPa.

The best combination of mechanical properties was obtained for a 2 mol.% Y_2_O_3_-stabilized ZrO_2_ composite with 20 wt.% WC, obtained by electroconsolidation at 1,350°C, combining a hardness of 16.5 GPa and a fracture toughness of 8.5 MPa m^1/2^.

## Competing interests

The authors declare that they have no competing interests.

## Authors' contributions

EG and OM were the principal investigators of this study. EG investigated the mechanical properties. OM investigated the structure and performed full factorial experiment for technology of hot pressing with direct transmission of high amperage current. VC prepared the experiment, carried out the X-ray analysis, and analyzed the results. All authors read and approved the final manuscript.

## References

[B1] Basu B Lee JH Kim DY Development of WC-ZrO_2_ nanocomposites by spark plasma sintering J Am Ceram Soc 2004 9 2 317 319 10.1111/j.1551-2916.2004.00317.x

[B2] Malek O Lauwers B Perez Y Baets P Vleugels J Processing of ultrafine ZrO_2_ toughened WC composites J Eur Ceram Soc 2009 9 16 3371 3378 10.1016/j.jeurceramsoc.2009.07.013

[B3] Pedzich Z Haberko K Piekarczyk J Faryna M Litynska L Zirconia matrix-tungsten carbide particulate composites manufactured by hot-pressing technique Mater Lett 1998 9 70 75 10.1016/S0167-577X(98)00010-X

[B4] Anstis GR Chantikul P Lawn BR Marshall DB A critical evaluation of indentation techniques for measuring fracture toughness: I. Direct crack measurements J Eur Ceram Soc 1981 9 533 10.1111/j.1151-2916.1981.tb10320.x

[B5] Lange FF Transformation-toughened ZrO_2_ correlations between grain size control and composition in the system ZrO_2_-Y_2_O_3_ J Am Ceram Soc 1986 9 3 40 242

[B6] Anné G Put S Vanmeensel K Jiang D Vleugels J Van der Biest O Hard, tough and strong ZrO_2_-WC composites from nanosized powders J Eur Ceram Soc 2005 9 1 55 63 10.1016/j.jeurceramsoc.2004.01.015

